# Supplementary material for: Fluid mechanics of the zebrafish embryonic heart trabeculation
Source: PLoS Comput Biol. 2022 Jun 6;18(6):e1010142. doi: 10.1371/journal.pcbi.1010142 (PMC9203006; doi:10.1371/journal.pcbi.1010142)
Supplement: S1 Text — (DOCX) [file pcbi.1010142.s001.docx]

**Supplementary Text for**

**Fluid Mechanics of the Zebrafish Embryonic Heart Trabeculation**

Adriana Gaia Cairelli^1^, Renee Wei-Yan Chow^2^, Julien Vermot^1,2^, Choon Hwai Yap^1^

*^1^ Department of Bioengineering, Imperial College London, London, United Kingdom*

*^2^ Institut de Génétique et de Biologie Moléculaire et Cellulaire (IGBMC), Strasbourg, France*

1. Zebrafish Embryo Preparation and Imaging Methods

Embryos at 3 days post fertilization (dpf) were treated with 0.003% 1-phenyl- 2-thiourea (PTU) (Sigma Aldrich) after 50% epiboly to prevent pigment formation. For some experiments, embryos were incubated with 4 μM BODIPY TR Ceramide (Thermo Fisher Scientific) for 20 minutes to stain the blood plasma for imaging. Before imaging, embryos were anesthetized with 0.02% tricaine (Sigma Aldrich) and mounted in 0.7% low melting-point agarose (Sigma Aldrich) in a glass-bottom petri dish (Matek). Realignment of the beating heart was performed post-imaging using BeatSync2.0.[1]

The resolution of the Leica DMi8 was 0.35μm in the x- and y-axes, and 0.61μm in the z-axis.

1. **Cardiac Motion Tracking Methods**

Cardiac motion tracking was performed using a validated cardiac motion estimation algorithm from our previous work,[2] which first performed pair-wise free-form deformation image registration, before regularizing the detected motions with a temporal Fourier and spatial b-splines motion model to ensure that calculated motions are cyclic and have spatial consistency. The calculated motion field enabled reanimation of a single reconstructed geometry to all time points, and as such, segment was only needed at one time point. The calculation motion field was also used in the CFD simulations.

1. Mesh Convergence Test and Mesh Density Analysis

Each individual inter-trabecular space was meshed with at least 1.3 million tetrahedral elements, in accordance to mesh convergence study from our past publication.[3]

The individual trabecular geometries were composed of at least 1.3 million tetrahedral elements, as determined by mesh convergence studies below. Three meshes were investigated in a representative ventricle, with 0.8 million, 1.3 million, and 2 million elements. Results in Fig A show that the wall shear stress (WSS) over the cardiac cycle was similar in all three meshes. The average error between the medium and fine mesh was found to be 0.035% for the WSS and 0.00001% for the velocity. For whole ventricular simulations, previous CFD simulations studies of the embryonic ventricle have shown that 0.8 million elements to be sufficient for mesh convergence.[4] In our study, we used at least 2.3 million tetrahedral elements for ventricular simulations, which should be sufficient.

| 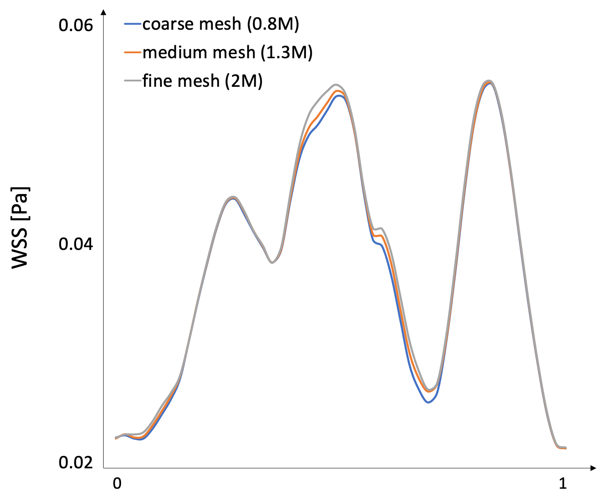 | Fig A – Mesh convergence test on WSS. Plot of WSS normalized over the cardiac cycle for different mesh size: a coarser mesh with 0.8 million elements, a medium mesh with 1.3 million elements, and a fine mesh with 2 million elements. |
| --- | --- |

1. CFD Simulation Methods

The fundamentals of CFD involves discretizing the fluid body into numerous mesh elements, and then solving the Navier-Stokes governing equation of fluid motion (essentially conservation of momentum and conservation of mass equations) for all the elements, to obtain the velocities and pressures at all nodes, from which other values such as WSS and OSI can be calculated. The software package used here, ANSYS fluent, solves the fluid dynamics via a finite volume approach, and is a widely used software package, as it has a wide range of features enabling robust and flexible simulation of varied scenarios. Details about the physical and computational theory involved in CFD modelling can be found in the ANSYS theory manual,[5] and in textbooks such as by Wendt et al.[6]

CFD Simulations were executed at 500 time steps per cardiac cycle for two cardiac cycles, and only the and this last cycle was used for analysis. This was sufficient as repeatability from the previous cycle was observed. The convergence criteria were specified to be less than 10^-4^ for all residuals.

From the CFD results, the spatial and temporal characteristics of WSS were analysed, and the oscillatory shear index (OSI) was calculated to investigate the extent by which wall shear stresses were oscillatory[7]:

$$OSI= \frac{1}{2}\left( 1-\frac{\left| \int_{0}^{T} \vec{\tau}dt \right|}{\int_{0}^{T} \left| \vec{\tau} \right| dt} \right)$$

where *T* is the cardiac cycle duration, *t* is time, and $\vec{\tau}$ is the WSS vector. The OSI ranges from 0 to 0.5, where zero means total unidirectional WSS from purely unsteady flow, and 0.5 signifies fully oscillatory flow with no net movement.

1. ***Various Scenarios used in the Inter-Trabecular Space Simulations***

To understand the importance of the interaction between the fluid in the inter-trabecular space with those in the main ventricular chamber, we conducted simulations where the individual inter-trabecular space was joined to the main ventricular chamber, while retaining the high mesh density in the inter-trabecular space, with full consideration of image-based motion dynamics of both chambers in the union, which is the “Baseline” scenario. The results were compared to simulations of the individual inter-trabecular space detached from the ventricle (with a zero-pressure boundary condition at the inlet of the space, which allowed fluid inflow and outflow but not fluid shear interaction at the boundary).

For individual intra-trabeculation space simulations, to determine whether translational and deformational motions of the endocardial boundary were important contributors to endocardial WSS, we simulated scenarios where either the translational motion or the deformational motions were removed (after the ventricle was detached from the inter-trabeculation space). Translation motions were estimated by the motion of the centroid of the walls, and were modelled as a Fourier equation over time for each of the three Cartesian coordinates. Deformational motions could be obtained by subtracting the translation motions from the original motions. Rigid body rotation motions were small, and as such were not removed.

Regarding the *fli1^+^/gata1^+^* cells in the inter-trabecular spaces, where the connections to the wall could be observed, they were modelled if they were part of the wall; while where such connections could not be observed, the cells were modelled as stand-alone structures within the fluid domain, akin to a Boolean subtraction from the fluid space. The surfaces of these cells were treated as impervious fluid boundaries, and were programmed to move during the CFD, according to the motion extracted from images, in the same way as how the endocardial walls were programmed.

1. Additional Results

Simulation Results for additional inter-trabecular spaces and whole ventricle models are presented below.


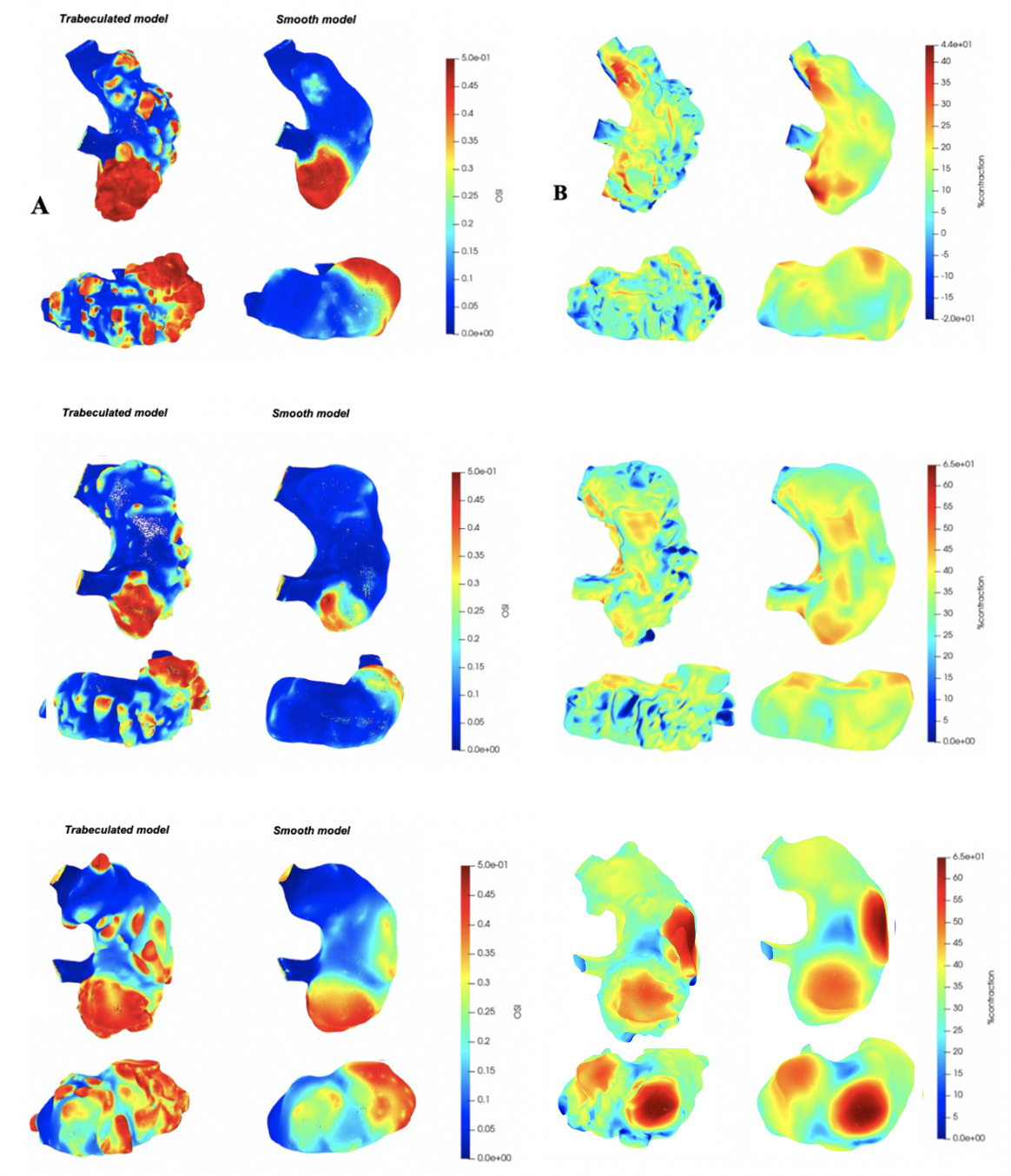


Fig B – OSI and endocardial contraction for the additional samples. Data to supplement Fig 6 C, D. Spatial pattern and surface-averaged magnitudes of (A) Oscillatory shear index (OSI) and (B) endocardial contractile surface area strains (end-diastole to end-systole) for both trabeculated and smoothed wall simulations of 3 additional zebrafish embryonic heart at 3dpf, at the lateral and ventral views.

Fig C – Endocardial WSS in the plasma viscosity scenario for the additional trabeculated and smooth samples. Whole ventricle CFD results for 3 additional embryos to supplement data in Figs 2A and 6B. (A) Contour maps of endocardial WSS over the cardiac cycle, with the assumption that fluid has the viscosity of plasma (1.5cP). Top row: ventral view of the outer curvature of the ventricle; bottom row: dorsal view of the inner curvature. (B) Contour maps of endocardial WSS in the smoothed version of the same ventricle, over the cardiac cycle with the assumption that fluid has the viscosity of plasma (1.5cP). Top row: ventral view of the outer curvature of the ventricle; bottom row: dorsal view of the inner curvature.


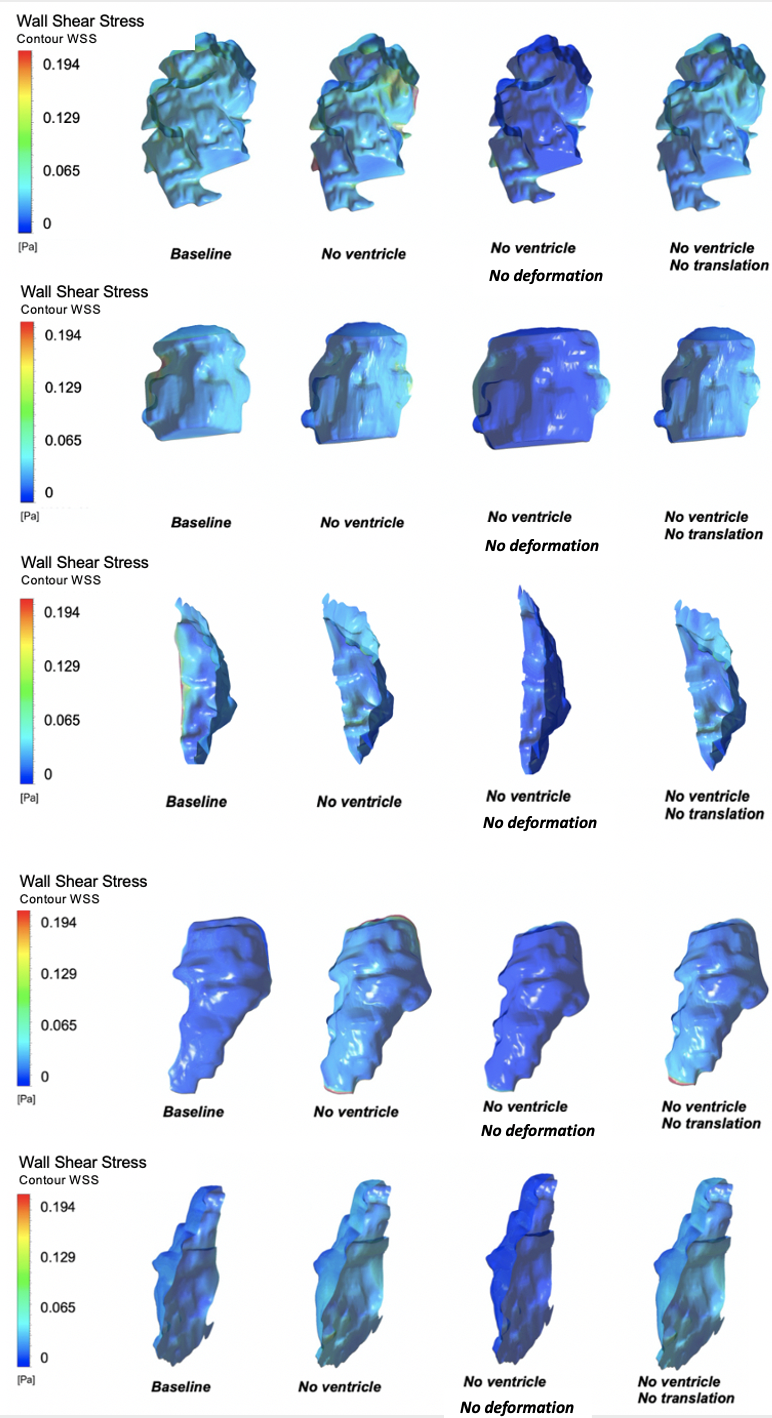


Fig D – Endocardial WSS under the various scenarios for the additional samples. CFD Results for another 5 intra-trabecular spaces to supplement results shown in Fig 4. End-systolic WSS under various scenarios.


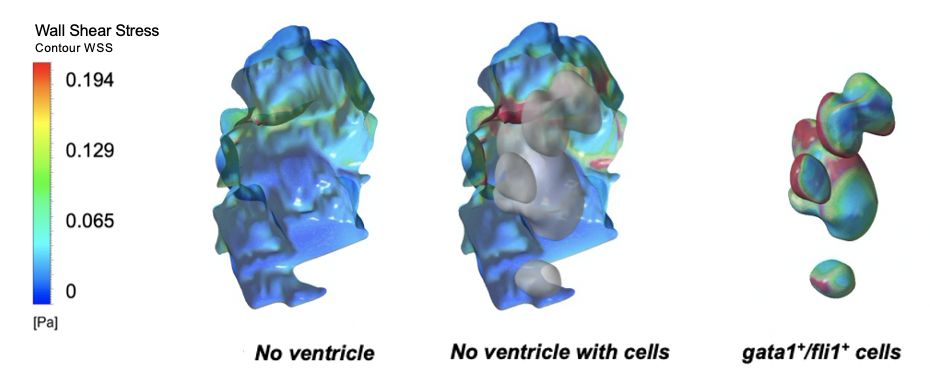


Fig E – Endocardial WSS under the effect of trapped cells for the additional sample. Flow simulation WSS results of another inter-trabecular space with and without hematopoietic cells at end-systole, to supplement data in Fig 5, demonstrating that the cells elevated endocardial WSS.


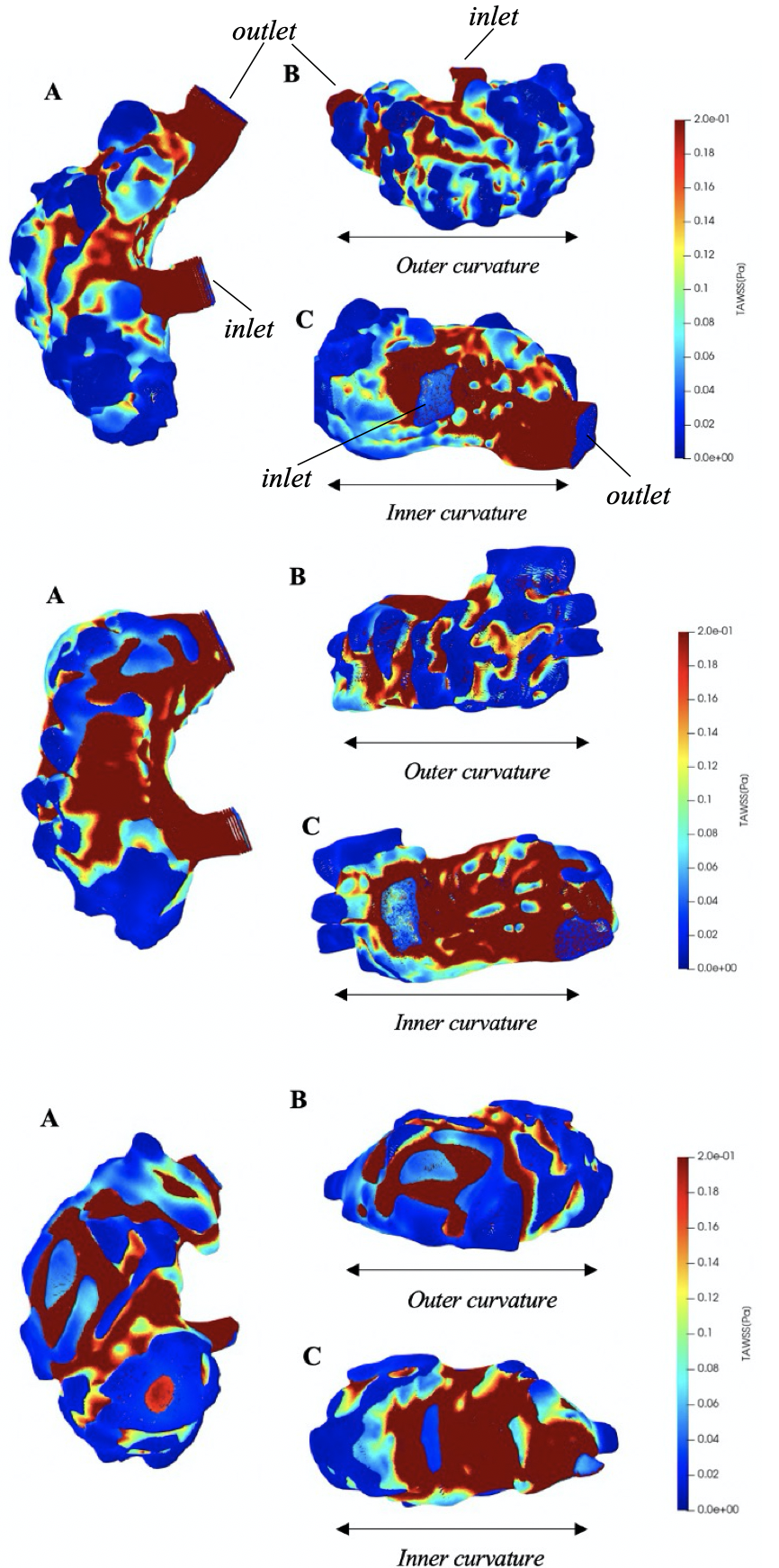


**Fig F – Endocardial WSS for the mixed-viscosity scenario for the additional samples. (A)** Contour map of the TAWSS for the mixed viscosity scenario in 3 additional embryonic hearts to supplement data in Fig 2B, **(B)** ventral view of the outer curvature and **(C)** dorsal view of the inner curvature. It is possible to see the distinction between the TAWSS on the grooves, estimated through the plasma viscosity and the TAWSS on the ridges, estimated through the blood viscosity.

**
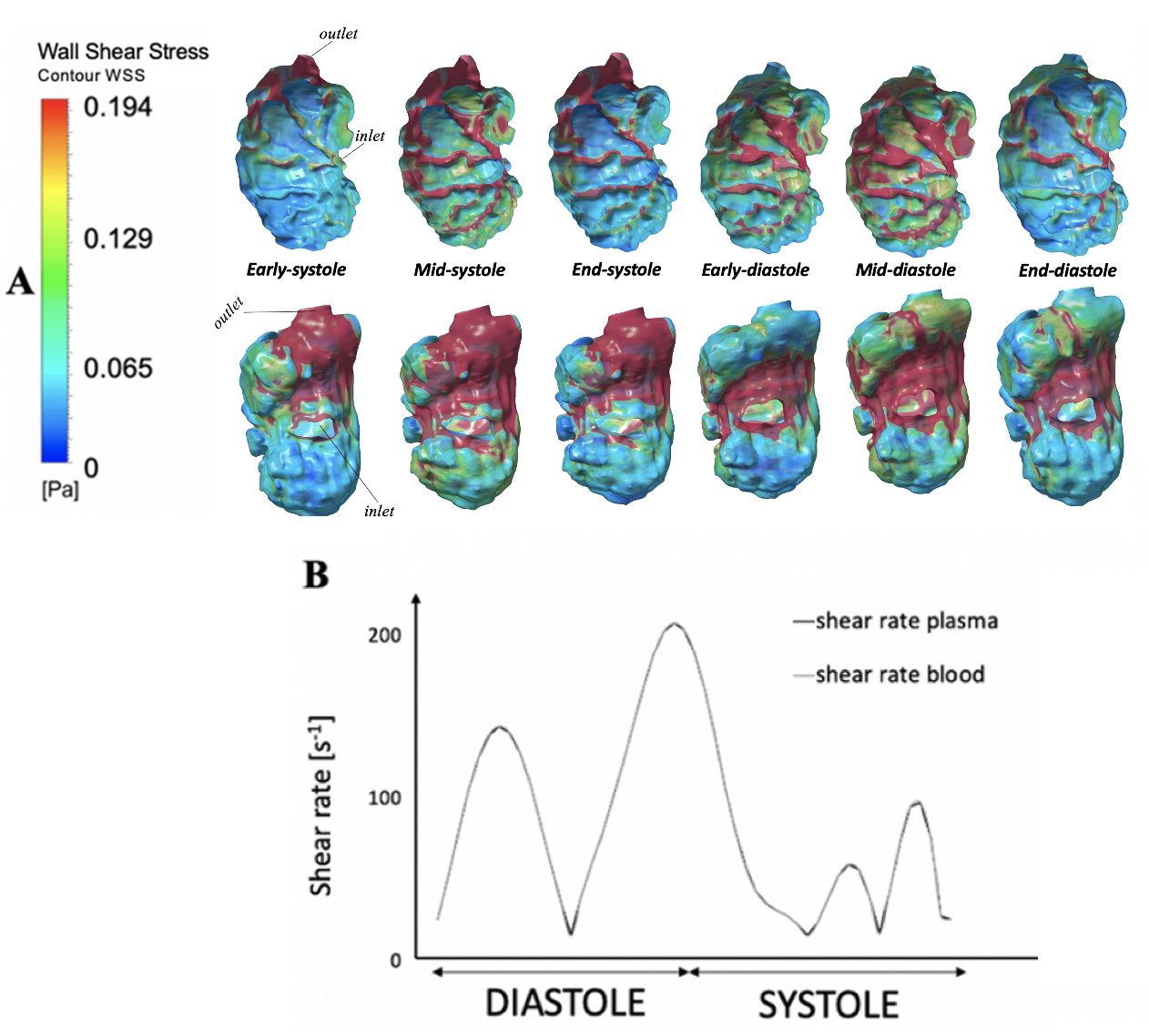
**

Fig G – Shear rate analysis and comparison between plasma and blood scenarios, and ridges and grooves structures. (A) Contour maps of endocardial WSS in a representative embryonic ventricle, over the cardiac cycle, with the assumption that fluid has the viscosity of blood (7.35 cP). Top row: ventral view of the outer curvature of the ventricle; bottom row: dorsal view of the inner curvature. (B) Wall shear rate in the same representative embryonic ventricle, over the cardiac cycle, for both the assumption that fluid has the viscosity of blood (7.35 cP) and of plasma (1.5 cP).


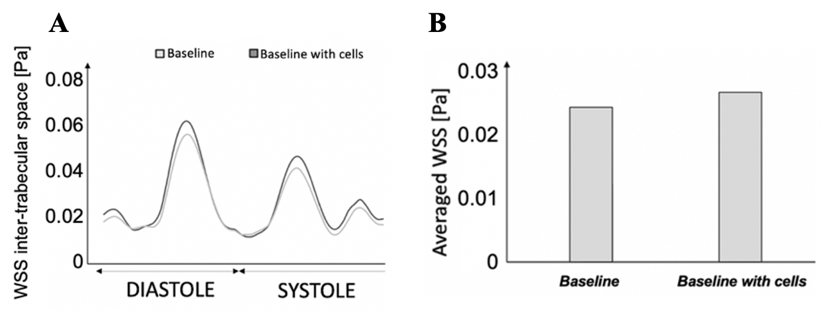


Fig H – Endocardial WSS to better capture the effect of trapped cells in the baseline scenario. Simulations of the inter-trabecular space connected to the whole ventricle (“Baseline” scenario), with and without the trapped cells within the inter-trabecular space, demonstrating that the presence of the trapped cells elevated endocardial WSS.

**REFERENCES**

1. Liebling M, Forouhar AS, Gharib M, Fraser SE, Dickinson ME. Four-dimensional cardiac imaging in living embryos via postacquisition synchronization of nongated slice sequences. J Biomed Opt. 2005;10(5):054001. Epub 2005/11/19. doi: 10.1117/1.2061567. PubMed PMID: 16292961.

2. Wiputra H, Chan WX, Foo YY, Ho S, Yap CH. Cardiac motion estimation from medical images: a regularisation framework applied on pairwise image registration displacement fields. Sci Rep. 2020;10(1):18510. Epub 2020/10/30. doi: 10.1038/s41598-020-75525-4. PubMed PMID: 33116206; PubMed Central PMCID: PMCPMC7595231.

3. Foo YY, Pant S, Tay HS, Imangali N, Chen N, Winkler C, et al. 4d modelling of fluid mechanics in the zebrafish embryonic heart. Biomech Model Mechanobiol. 2020;19(1):221-32.

4. Foo YY, Pant S, Tay HS, Imangali N, Chen N, Winkler C, et al. 4D modelling of fluid mechanics in the zebrafish embryonic heart. Biomech Model Mechanobiol. 2020;19(1):221-32. Epub 2019/08/26. doi: 10.1007/s10237-019-01205-6. PubMed PMID: 31446522.

5. Manual U. ANSYS FLUENT 12.0. Theory Guide. 2009.

6. Wendt JF. Computational fluid dynamics: an introduction: Springer Science & Business Media; 2008.

7. He X, Ku DN. Pulsatile flow in the human left coronary artery bifurcation: average conditions. J Biomech Eng. 1996;118(1):74-82. Epub 1996/02/01. doi: 10.1115/1.2795948. PubMed PMID: 8833077.
