# Supplementary material for: Fluid mechanics of the zebrafish embryonic heart trabeculation
Source: PLoS Comput Biol. 2022 Jun 6;18(6):e1010142. doi: 10.1371/journal.pcbi.1010142 (PMC9203006; doi:10.1371/journal.pcbi.1010142)
Supplement: Attachment — Submitted filename: Rebuttal Letter PLOS_v5.docx [file pcbi.1010142.s006.docx]

**Response to Review Comments**

***Reviewer #1***

General comments:

The authors simulated the blood flow of 3dpf embryonic zebrafish heart to elucidate the effect of ventricular geometry. Their results were improved from previous research by including endocardial layers which is direct contact boundary of blood. Although WSS increased after including endocardial layers (~10um thick), interestingly, WSS pattern remain similar which could validate their simulation results. In addition, they tried to understand what causes the driving flow and WSS of the inter-trabecular spaces. They simulated the different scenarios of geometry of inter-trabeculae and found that the inter-trabecular squeeze flow effect is the main driver and translational motion of space doesn’t significantly contribute to the flow and WSS. Another interestingly finding is that their endothelial-to-hematopoietic transition could happened in inter-trabeculae regions. Based on their spinning disk confocal, they observed cells that positively expressed both fli1 and gata1. Overall, this research could provide an important direction of the mechanotransduction in cardiac development using zebrafish. However, there are some concern should be address.

***Response:*** We thank the reviewer for the constructive comments that has helped us improve our manuscript. We address them below.

1. Authors measured the dimensions of zebrafish trabeculae as longitudinal, radial and circumferential dimensions were 12.5±3.1 μm, 8.7±0.8 μm, 21.7±10.7 μm (n=16) respectively. However, it is unclear where the author measured. Trabeculae mostly develop in outer curvature, but some are near apex and outflow tract. Trabeculae near outflow tract and apex should be relatively small. It would be helpful to address specifically where the author measured. Also, the measurement are in relaxation stage or contraction stage? Also, if you can make a table, it would be helpful to present.

***Response:*** We thank the reviewer for the suggestion. We measured the trabeculae on the outer curvature at several different locations (n=16), including near the apex and the outflow tract. The reviewer is right, some trabeculae were smaller than others. For this reason, we have surveyed many inter-trabecular spaces, and tried to include trabeculae with different shapes and sizes and from different locations to have a good sense of their dimensions. The measures were all taken at the same time point in the cardiac cycle, at the end of the systole. We added this information in line 123-124. However, since we are only reporting 3 types of measurements, we think it can be left in the text rather than making a new table.

2. Authors mentioned in line 177-180 that high WSS found at inner curvature compared to outer curvature due to low Reynolds numbers. Although he properly cited, it would be helpful to briefly explain what makes this event.

***Response:*** At higher Reynolds number, fluid momentum is stronger than viscous forces, and fluid momentum tend to retain the initial direction of flow as it enters the curved channel, causing velocities to be higher at the outer curvature and lower at the inner curvature. Consequently, wall shear stresses are higher at the outer curvature. On the other hand, with low Reynolds number, viscous forces are much stronger than fluid momentum, and flow directions are easily bent by adverse pressure gradient in a curved channel. This results in velocities to be higher at the inner curvature and lower at the outer curvature instead, and consequently, wall shear stresses are higher at the inner curvature. This skew in flow profile towards the inner curvature has been demonstrated numerically [1], and the higher inner curvature WSS has been demonstrated experimentally [2]. We added text in lines 181-186 to briefly explain this phenomenon.

3. Authors should describe better how to simulate hybrid approach to calculate WSS of inter-trabeculae where blood cells can’t occupy while heartbeat. It is interesting approach without using two phase flow simulation. However, detail explain should be address in methods section

***Response:*** We thank the reviewer for the comment, and we agree that more details are needed. We clarify that we did not simulate both viscosities in the same simulation, but a simulation was conducted for each viscosity scenario. Next, WSS results for the trabeculae region were obtained from the plasma viscosity simulation, while WSS results for the non-trabeculae region were obtained from the blood viscosity simulation, and they were combined to give the hybrid scenario. We added text in lines 191-195 to explain this.

4. Direction of zebrafish heart in Fig. 2 and supplementary figures are not well depicted. For example, for Fig. 2A, outlets of the upper figures are pointing left side, but lower figures are opposite. Zebrafish heart directions should be consistent and clear. For supplementary figures, author didn’t point out where the outflow tract and inlet flow are. Thus, it took me a time to figure out where the area between inlet and apex.

***Response:*** We optimized all relevant figures (Fig. 2A, 2B, 6B, S3, and S7): (1) re-oriented the hearts to maximize them to be more visible, (2) orienting all hearts to point in the same direction (outlet at the top), (3) added labels of where the inlets and outlets are to avoid confusion. This should now be much better, but please let us know if this can be further improved.

***Reviewer #2***

General comments:

In this study, the authors use computational fluid dynamics to analyze the fluid mechanics of trabeculations in developing zebrafish. Using a fish line that expresses endocardial lining, the study finds that trabeculations lead to a spatially varying and oscillatory shear profile. This is further attributed to a local squeezing flow that has minimal interaction with the bulk flow in the ventricular cavity.

The analysis is interesting and the manuscript is generally well written with some minor typos and sentence construction errors that could be rectified during revision. However, the quality of the manuscript could be substantially improved by furnishing additional details and clarifying some areas to avoid confusing the reader and facilitate reproducibility.

***Response:*** We thank the reviewer for the constructive comments that has helped us improve our manuscript. We address them below.

Major:

- One aspect that is ignored in this study is that trabeculations form a network. During deformation, the network could undergo `squeezing` motion as rightly predicted by the authors and also hypothesized by Ares Pasipoularides (Heart’s Vortex). This complex motion could lead to contacting surfaces and may provide additional contribution to stroke volume. In this study, however, the endocardial lining is extracted as a continuous surface that remains intact during the deformation. The complex squeezing motion of the trabeculations with contact is nearly impossible to model but should be addressed as a limitation of the approach.

***Response:*** We thank the reviewer for raising this interesting point. From our images, the trabeculation structures in these 3dpf zebrafish embryonic hearts were immature and were not as complex as in adult hearts. They appear as individual pockets instead of a network of inter-connected channels. However, we agree that there could be occurrences of endocardial lining folding that our image motion tracking algorithm could not capture, and as such folded surfaces would be modelled as a continuous but smaller surface, rather than a truly folded surface. We added limitations text on this in lines 514-518.

We also thank the reviewer for bringing up this interesting book. We agree that the squeezing motion of the trabeculation likely contributes to cardiac function, but we think that this may need deeper studies to fully understand. In terms of contributing to the stroke volume, since the inter-trabecular spaces are small in volume compared to the main chamber, their cumulative contribution to stroke volume was small (5-8% of the total stroke volume). We think that the more important contribution of the trabeculation towards heart function may be via tissue mechanics rather than fluid mechanics, where their structure and location allow them to execute contractile motion and generate contractile forces more easily, but further work is needed to properly investigate this.

- Page 4, Lines 92-95; Page 5, Line 110: It is not clear what the authors mean by optimized estimation of endocardial WSS. This needs to be clarified or rewritten.

Perhaps the focus could be on the mechanism for generating fluid forces in the intra- and inter-trabecular spaces?

***Response:*** We replaced “optimized” with “improved” to clarify the statement (lines 109-110):

“In the current study, we sought to build upon these existing works and achieve an improved quantification of the zebrafish embryonic endocardial flow stresses.”

- WSS characterization (Page 7, Lines 150-155): It is noted that the WSS for simulations with different viscosities can be obtained by simply scaling with the corresponding ratio, and this is attributed to the low Re. However, this could also be attributed to the flow being assumed to be Newtonian and therefore, viscosity is independent of shear rate.

While this is interesting and could avoid the need to perform complex simulations involving blood rheology, can the authors provide the range of shear rates observed in the ventricle? Does assuming non-Newtonian flow change the viscosity substantially at low shear rates? If so, then simple scaling of WSS due to change in viscosity may not be possible.

***Response:*** We thank the reviewer for being cautious. From our microscopy movies, it can be observed that flow in the hearts are typically quite dynamics. Further, we showed in Fig. S7B the range of spatial-averaged shear rates expected in the ventricle for simulations with both plasma and blood viscosity assumptions, and it could be observed that shear rates were in the range of 20-200s^-1^ over the cardiac cycle. Within this range, blood viscosity does not typically change much [3] (figure R1 below), thus the Newtonian fluid assumption should be sufficiently accurate even though we agree that it was an idealization.

Figure R1 – Viscosity trend with respect to shear strain rate under different blood viscosity models assumptions. Adapted from [3]

- Inter-trabecular analysis (Page 11, Lines 225-232): The setup of analyzing individual trabeculations is intriguing, especially the zero-pressure boundary condition. This boundary condition would not capture shearing motion between the bulk ventricular flow and the flow in the trabecular spaces.

Further, when the trabeculations expand and contract, they would exchange flow with the ventricular cavity. Therefore, it is misleading to suggest that the interaction is not necessary to generate inter-trabecular flow (lines 230-232).

More details need to be furnished on how the isolated trabeculations simulations are set up. How is the ventricle detached or attached to the trabeculations? Where are the boundary conditions applied? Is there no flow at all in the bulk cavity in the no-ventricle case? Are the individual trabeculations solved?

For the sake of completeness, can the authors perform analysis on OSI for the individual trabeculations?

***Response:*** We agree that it is misleading to claim that no interaction with the main chamber is necessary to generate the trabecular flow, given that for the individual trabecular space simulation, there is still flow going in and out of the zero-pressure boundary at the inlet of the trabecular space. As such, we modified the claim to be that no **shear** interaction with the main chamber was necessary to generate trabecular flow. (line 250, 255 and also abstract line 40 and conclusion line 440-441)

We clarify the single trabecular space simulation methods here. The single trabecular space was detached from the ventricle by cropping the ventricular out along a single plane close to the inlet of the trabecular space. This was conducted manually. The opening of the trabecular space left behind by the cropping is then specified as reference zero pressure boundary condition. Simulations were then conducted just for the single trabeculation space, independent of the main ventricular space (because flow in the main ventricle is not relevant anymore after the detachment). The reviewer is right that this will still allow flow into and out of the trabecular space, but it will not include shear interactions between the main chamber and the trabecular fluid space. We clarified these details to methods section (line 568-580).

Regarding the OSI for the single intra-trabecular spaces, we provided the average of OSI for these trabecular groove spaces in Figure 3C, to show that they were higher than OSI at the trabeculation ridges. We also showed the spatial variability of OSI on the whole trabeculated ventricle in Figure 6C, again to show that OSI were higher in the trabeculations grooves than ridges. We think that OSI is best characterized with the whole ventricular simulations. The individual trabeculation space simulations were intended to discover the main driver of flow and WSS in these spaces, and not for characterizing OSI. We hope the reviewer will agree with this.

Figure R2 – On the left, OSI with a focus on the intra-trabecular spaces (corresponding to Figure 6C). On the right, Fig. 3C extracted from the manuscript.

- On the effect of cells in the inter-trabecular spaces, it is not clear how these cells are included/excluded in the analysis. Are they introduced in the model as finite obstructions that are connected to the outer wall? Are they individually tracked?

***Response:*** We first clarify that our motion tracking algorithm uses a whole-field approach, where motion anywhere in the field of view is described, via a b-spline spatial interpolation of temporal Fourier coefficients at grid points. Therefore, all features within the 3D field of view could be tracked. Our previous publication on the tracking algorithm can explain this in details [4]. As such, all these cells, as far as could be observed in the image, were tracked. Where the connections to the wall could be observed, the cells were modelled if they were part of the wall. Where such connections could not be observed, the cells were modelled as stand-alone “islands” within the fluid domain, akin to a Boolean subtraction from the fluid space. The surfaces of these cells were treated as impervious fluid boundaries, and were programmed to move during the CFD, according to the motion extracted from images, in the same way as how the endocardial walls were programmed. We added text in supplementary text in lines 88-94 to clarify these methods.

- On page 17, line 335, it is conjectured that recirculatory flow could be observed in the inter-trabecular spaces. As the authors here perform flow analysis in the individual trabeculations, can the authors use data to support this argument? IS the flow recirculatory in these cavities?

Is the higher OSI in these cavities attributed to the recirculatory flow or the changing flow direction between filling and ejection?

***Response:*** We thank the reviewer for the comment. We believe that the high OSI is attributed to the changing flow direction between filling and ejection, as we could not see any significant vortex formations were observed in the middle of the grooves as shown in fig. R2 below. We added text in line 360-363 to include these discussions.

Figure R3 - Vorticity color contour within a 2D cut-plane of the groove space for the mid-diastolic and mid-systolic period. No significant vortex formations were observed in the middle of the groove.

- In the Methods section, can more details be added on how the images scanned were synchronized to the cardiac cycle? Is the imaging volumetric or was scanning performed on a single plane for a certain time before advancing to the next plane?

***Response:*** The images were acquired in XYTZ mode (see line 530, so the scanning was performed on a single plane for a certain fixed time before advancing to the next plane. The realignment and synchronization of the image slices was conducted using BeatSync2.0, using methods reported by Liebling et al.[5]. We added text in in the supplementary text (lines 16-17) to guide readers.

- On the Data Availability, while the main CFD solver commercial and could not be shared publicly. However, the authors could provide access to images, ventricular models, any codes used to process the images and extract motion, any post-processing scripts, etc. Otherwise, this would seriously affect reproducibility of the results. Any restrictions on the data/code availability should be clearly specified in the manuscript.

***Response:*** We thank the author for raising this important point. As the 4D image and simulation files are quite big, we will share them upon request rather than upload them, but we can provide a sample of the ANSYS user defined function that was used for simulations as a supplementary file. The motion tracking code is already available in github as ’WeiXuanChan/motionSegmentation’, as reported in our previous publication [4]. We added text in line 597-600 to inform readers of these, and hope these are acceptable to the reviewer

Minor:

- Please thoroughly revise the manuscript to fix some minor spelling mistakes and sentence constructions errors.

***Response:*** We vetted and fixed all errors that we can find.

- Abstract: something is missing in the sentence, "By comparing our results to literature..." please rectify.

***Response:*** We rectified this sentence to be “Our endocardial wall shear stress (WSS) results were found to exceeded those reported in existing literature, which were estimated using myocardial rather than endocardial boundaries.”

- Can the authors cite refs 9 and 18 wherever spatial variability in WSS is discussed to support their arguments? Also, some references have co-first authors. Please rectify.

***Response:*** We thank the reviewer for the comment. We will cite refs 9 and 18 wherever spatial variability in WSS is discussed.

- The authors generally refer to `inter-trabecular' spaces in the manuscript for identifying the regions within the trabecular cavities. However, it might be better to use the word `intra-trabecular' to refer to these cavities and reserve `inter-trabecular' to the endocardial segment between the trabeculations.

***Response:*** We agree and have rectified this.

- Fig 6C, 6D. please provide x-axis labels/legends for the bar charts.

***Response:*** We added this.

- Page 20, lines 397-399, it is mentioned that blood viscosity is locally varied between trabecular ridges and in the inter-trabecular spaces. Can the authors confirm if the viscosity is spatially varied in the problem setup? Can this be highlighted in the Methods section?

***Response:*** We clarify that the hybrid scenario was not conducted with one simulation. Two simulations with two different viscosities were conducted, and then the results of the intra-trabecular fluid space was obtained from the simulation with plasma viscosity, while the results in all other fluid spaces were obtained from the simulation with blood viscosity, and these two results were combined to give the results of the hybrid scenario. We clarified this methodology in line 191-195.

***Reviewer #3***

General comments:

This paper demonstrates a CFD-based shear stress analysis in embryonic zebrafish heart. Compared to most of previous studies, it adopts high resolution spinning-disk confocal microscopy and fish lines that label the endocardial cell membranes rather than myocardial cells. The ventricular domain, as well as the trabeculation ridges and grooves could therefore be defined in a more accurate fashion. The novel aspects are using an endocardial marker to better resolve trabecular structures, and the interesting result that squeezing motion is the main driver of flow in inter-trabecular spaces. Overall, the paper is interesting, although the methods do not appear to be particularly novel, and the analysis is fairly simple on the fluid mechanics side.

***Response:*** We thank the reviewer for the in-depth comments and guidance. Indeed, we have followed excellent methodologies published previously by Lee, Vedula, Marsden, Hsiai and co-workers, and seek to contribute by reporting novel findings using the endocardial marker line. We address reviewer’s comments as follows.

Major Comments

1. The use of the “mixed viscosity” model needs better justification. In the Stokes flow limit, structures in a flow have a long-ranged effect. Even though the red blood cells are not in the inter-trabecular spaces, they may still have a strong effect on the fluid mechanics in these spaces. In addition, the bulk viscosity assumption for blood is only valid when the length scale of the flow is much greater than the length scale of the blood cells. From the authors’ movies, the size of the blood cells is fairly large compared to the ventricle itself and is certainly on a similar scale to the inter-trabecular spaces. Uncertain is the bulk viscosity assumption, and further clarification is needed in the discussion.

a. The authors may consider performing a fluid-structure interaction simulation with fully-resolved RBCs to support the result with that of bulk viscosity assumption.

***Response:*** We thank the reviewer for this important point. We agree that only with two-phase simulations with fully-resolved RBC biomechanics can the wall shear stresses be very accurately obtained. We have not developed this methodology, but hope to do so in future. We have limitations text to acknowledge that our modelling of fluid as a continuum rather than 2-phase particulate fluid is a limitation (lines 506-509), we toned down our claim of the mixed-viscosity model in lines 198-200, and we added text to acknowledge that the best WSS estimates can only be done with the 2-phase simulations with RBC biomechanics (lines 433-436)

However, we believe that the single-phase CFD simulations can still achieve a good estimation on the order of magnitude of WSS, and temporal patterns of WSS, and effects of trabecular squeezing motion. Previous studies using single-phase CFD simulations have provided us with much understanding of the embryonic heart fluid mechanics [6-9]. We think that with single-phase simulations, our mixed-viscosity approach would be a good way to move closer to the WSS truths.

Regarding the Stokes flow, we agree that the viscous effects in the main chamber may affect the fluid forces in the trabecular grooves under this theory. However, we managed to check this effect with our “Baseline” versus “No Ventricle” simulations. As we have shown in Fig. 4B and 4C, although the effects of the flow in the main chamber on the grooves had an impact on the fluid force patterns, they do not affect the magnitude of the fluid forces much. We thus think that the stokes effect of viscosity in the main ventricle would not affect flow and WSS in the intra-trabeculation spaces much.

2. Line 351: This section about endocardial deformation is interesting, and further development or removal from the discussion as the manuscript seems to be primarily focusing on fluid mechanics.

a. The authors note that the surface strains were smaller in the trabeculated heart than the non-trabeculated heart. Isn’t this due to the higher surface area in the trabeculated than non-trabeculated heart?

b. Is there any spatial variability in surface strains? Higher/lower in the trabecular ridges/grooves?

***Response:*** On our results that trabeculated ventricle need less surface strain to achieve the same stroke volume as the smooth ventricle, we think this is due to the surface topology rather than surface area.

We first clarify that the strain we quantified is a quantity that is independent of surface area magnitude. It is calculated by normalizing a measure of the change in length by the original length, and is expressed as a percentage. Since it is normalized and dimensionless, it is independent of the surface area magnitude.

Rather, we think that because the trabeculated heart has a corrugated surface geometry, the surface strains needed to achieve a specific stroke volume were lower. This was because a corrugated surface could rely on shape changes such as straightening out or becoming more creased to bring about cardiac motions, and these shape changes could be achieved without much strain to its actual surface. Eg., there is no need to stretch the surface when we are simply straightening out a corrugated surface to increase ventricular volume. We added text in line 385-389 to explain this in the manuscript.

From our results, it looks like surface strains are higher in the grooves and lower in the ridges, so yes, we do observe spatial variability in strains. This can be observed from figure 6D.

Minor Comments

1. The authors may edit and address multiple typos.

***Response:*** We further vetted the manuscript and corrected all errors found.

2. Line 121: Please provide a schematic to explain the relevant geometry to the “inter-trabecular space”.

***Response:*** We thank the reviewer for the comment. We added the schematic as Fig. 1C to guide readers.

3. Figures 1, 2: Images are too small. It is difficult to see the important features. Seeing your supplementary movies, the full-size renderings are quite striking to see, so I would like to see them in the paper.

***Response:*** We thank the reviewer for the comment.

4. Line 143: In this section, define WWS and wall shear rate.

***Response:*** We defined the acronym WSS in line 92, where it was used the first time. We defined wall shear rate in line 150 as suggested.

5. Line 222: In this section, please explain in detail the different scenario and how they are defined in the methods.

***Response:*** We prepared detailed explanations of the various scenarios in supplementary text section 5. We also prepared text in figure caption of figure 4 to explain the various scenarios, to supplement the explanations in the section at lines 231-268.

6. Line 251: In the case where translational motions were removed, were rigid body rotations also removed? Typically, motions are decomposed into translation, rotation, and deformation.

***Response:*** We thank the reviewer for raising this. Indeed, rotation are not removed when translation was removed. However, rigid body rotation motions were small, with a maximum of approximately 0.07 rad over the whole cardiac cycle (with the end-diastole as the reference point). We added text in lines 266-268 to explain this.

7. Line 261: The cells may be “attached” to the endocardial surface via lubrication forces.

***Response:*** This is an interesting proposal, but we think that this is not likely, because the cells reverted to their initial locations with high precision at the end of 1 cardiac cycle, and displayed very little randomness. If they were held solely by lubrication forces, it seems that more randomness should be expected. Nonetheless, we added text at lines 278-283 to discuss this notion and put forth our views, so that readers to make their own interpretation.

8. Line 476: Please further discuss the limitations; that is, estimate how your approximations and assumptions affect the validity of your answer.

***Response:*** We added more details to this section to explain the effects of our limitations. Essentially, the WSS will have more temporal unsteadiness, as they will be higher when an RBC was passing by, but lower when there was none. However, we expect that the ensemble averaged or smoothed out WSS waveforms to be close to our current results. Just as the reviewer has pointed out this affects not just the non-trabeculated regions and trabeculation ridges, but it can also affect the inter-trabecular spaces due to stokes flow effects, but it is not yet clear how strong these effects are.

9. Line 507: Have you validated the motion tracking algorithm with this data by comparing the reconstructed geometry to manual segmentations at later time points in the cardiac cycle?

***Response:*** We thank the reviewer for the comment. Our motion tracking algorithm was already validated in a previous study, and is published [4]. We tested our algorithm with a publicly available data from the cardiac motion analysis challenge, which has echo images of adult hearts with MRI truths, and found that our algorithm was 10% more accurate than contemporary methods used the participants of the competition, in terms of the surface Euclidean distance error. We also validated the technique with manual segmentation, and found good matches in all our tests.

**References:**

1.	Pantokratoras A. Steady laminar flow in a 90° bend. Advances in Mechanical Engineering. 2016. doi: <https://doi.org/10.1177/1687814016669472>.

2.	Peterson LM, Jenkins MW, Gu S, Barwick L, Watanabe M, Rollins AM. 4D shear stress maps of the developing heart using Doppler optical coherence tomography. Biomed Opt Express. 2012;3(11):3022-32. Epub 2012/11/20. doi: 10.1364/BOE.3.003022. PubMed PMID: 23162737; PubMed Central PMCID: PMCPMC3493225.

3.	Liu H, Lan L, Abrigo J, Ip HL, Soo Y, Zheng D, et al. Comparison of Newtonian and Non-newtonian Fluid Models in Blood Flow Simulation in Patients With Intracranial Arterial Stenosis. Front Physiol. 2021;12:718540. Epub 20210906. doi: 10.3389/fphys.2021.718540. PubMed PMID: 34552505; PubMed Central PMCID: PMCPMC8450390.

4.	Wiputra H, Chan WX, Foo YY, Ho S, Yap CH. Cardiac motion estimation from medical images: a regularisation framework applied on pairwise image registration displacement fields. Sci Rep. 2020;10(1):18510. Epub 2020/10/30. doi: 10.1038/s41598-020-75525-4. PubMed PMID: 33116206; PubMed Central PMCID: PMCPMC7595231.

5.	Liebling M, Forouhar AS, Gharib M, Fraser SE, Dickinson ME. Four-dimensional cardiac imaging in living embryos via postacquisition synchronization of nongated slice sequences. J Biomed Opt. 2005;10(5):054001. Epub 2005/11/19. doi: 10.1117/1.2061567. PubMed PMID: 16292961.

6.	Foo YY, Pant S, Tay HS, Imangali N, Chen N, Winkler C, et al. 4D modelling of fluid mechanics in the zebrafish embryonic heart. Biomech Model Mechanobiol. 2020;19(1):221-32. Epub 2019/08/26. doi: 10.1007/s10237-019-01205-6. PubMed PMID: 31446522.

7.	Lee J, Fei P, Packard RR, Kang H, Xu H, Baek KI, et al. 4-Dimensional light-sheet microscopy to elucidate shear stress modulation of cardiac trabeculation. J Clin Invest. 2016;126(5):1679-90. Epub 2016/03/29. doi: 10.1172/JCI83496. PubMed PMID: 27018592; PubMed Central PMCID: PMCPMC4855946.

8.	Lee J, Moghadam ME, Kung E, Cao H, Beebe T, Miller Y, et al. Moving domain computational fluid dynamics to interface with an embryonic model of cardiac morphogenesis. PLoS One. 2013;8(8):e72924. Epub 2013/09/07. doi: 10.1371/journal.pone.0072924. PubMed PMID: 24009714; PubMed Central PMCID: PMCPMC3751826.

9.	Lee J, Vedula V, Baek KI, Chen J, Hsu JJ, Ding Y, et al. Spatial and temporal variations in hemodynamic forces initiate cardiac trabeculation. JCI Insight. 2018;3(13). Epub 2018/07/13. doi: 10.1172/jci.insight.96672. PubMed PMID: 29997298; PubMed Central PMCID: PMCPMC6124527.
